# Conserved function of *Drosophila* Fancd2 monoubiquitination in response to double-strand DNA breaks

**DOI:** 10.1093/g3journal/jkac129

**Published:** 2022-05-20

**Authors:** Delisa E Clay, Erin A Jezuit, Ruth A Montague, Donald T Fox

**Affiliations:** Department of Pharmacology and Cancer Biology, C318 Levine Science Research Center, Duke University Medical School, Durham, NC 27710, USA; Department of Pharmacology and Cancer Biology, C318 Levine Science Research Center, Duke University Medical School, Durham, NC 27710, USA; Department of Pharmacology and Cancer Biology, C318 Levine Science Research Center, Duke University Medical School, Durham, NC 27710, USA; Department of Pharmacology and Cancer Biology, C318 Levine Science Research Center, Duke University Medical School, Durham, NC 27710, USA

**Keywords:** *Drosophila*, Fancd2, double-strand break, radiation, monoubiquitination, Mre11

## Abstract

Fanconi anemia genes play key roles in metazoan DNA damage responses, and human FA mutations cause numerous disease phenotypes. In human cells, activating monoubiquitination of the Fanconi anemia protein Fancd2 occurs following diverse DNA damage stimuli. Monoubiquitinated Fancd2 forms nuclear foci to recruit additional repair factors. Fancd2 animal models to date have focused on molecular nulls or whole gene knockdown, leaving the specific in vivo role of monoubiquitination unclear. Using a point mutant in a conserved residue, we recently linked *Drosophila* Fancd2 monoubiquitination to a mitosis-specific DNA double-strand break response. In this context, we used CRISPR/Cas9 to generate the first animal model of an endogenous mutation in the conserved monoubiquitination site (*fancd2*^K595R^). Here, we expand upon our characterization of *fancd2*^K595R^. We also introduce and characterize additional *Drosophila* tools to study *fancd2*, including new mutant alleles and GFP-tagged rescue transgenes. Using these new reagents, we show the impact of *Drosophila* Fancd2 on organismal and cell viability, as well as on repair protein localization, in the presence or absence of double-strand breaks. These findings expand our understanding of Fanconi anemia gene function in vivo and provide useful reagents for DNA repair research.

## Introduction

Human Fanconi anemia (FA) is an autosomal recessive condition, first identified by Guido Fanconi (Lichtman et al. 2000). This disease is characterized by bone marrow failure and cancer predisposition, with several cancer types that disproportionally impact children (Mehta *et al.* 2002; Fang *et al.* 2020). FA patient cells fail to resolve multiple DNA lesions, such as inter-strand cross links (ICLs), replicative stress, and double-strand breaks (DSBs). FA cells are therefore sensitive to ICL agents, sources of replication stress, and DSB agents such as X-rays (Chan *et al.* 2009; Leskovac *et al.* 2014; Rodríguez and D’Andrea 2017; Niraj *et al.* 2019; Zahnreich *et al.* 2020).

FA genes perform distinct functions as part of a molecular pathway. The FA core complex includes FancL, a ubiquitin ligase (Hodson *et al.* 2011; Ceccaldi *et al.* 2016; van Twest *et al.* 2017; Liu *et al.* 2020). This complex monoubiquitinates the proteins Fancd2 and FancI, which form a heterodimer (Sims *et al.* 2007; Smogorzewska *et al.* 2007; Joo *et al.* 2011; Rajendra *et al.* 2014; Ceccaldi *et al.* 2016; Rodríguez and D’Andrea 2017; Niraj *et al.* 2019; Shakeel *et al.* 2019; Lemonidis *et al.* 2021; Wang *et al.* 2021). Monoubiquitination of Fancd2 and FancI are thought to be the activating step of the pathway. Ubiquitinated Fancd2 and FancI localize to nuclear repair foci following DNA damage (Garcia-Higuera *et al.* 2001; Sims *et al.* 2007; Smogorzewska *et al.* 2007; Chan *et al.* 2009). These foci are sites for recruiting downstream effector proteins that are involved in various replication, repair, and cell cycle processes (Moldovan and D’Andrea 2009; Smogorzewska *et al.* 2010; Yamamoto *et al.* 2011; Chaudhury *et al.* 2014; Ceccaldi *et al.* 2016; Lachaud *et al.* 2016; Rodríguez and D’Andrea 2017; Niraj *et al.* 2019; Liu *et al.* 2020).

Orthologs of several FA genes including Fancd2 are found in several model organisms. To date, studies of Fancd2 in these organisms have focused on molecular null or whole gene knockdown alleles. These studies have linked Fancd2 to organismal viability, germ cell function, and cancer predisposition in the absence of DNA damage. Further, mirroring findings in human cells, Fancd2 mutants in mice, zebrafish, *Caenorhabditis* *elegans*, and *Drosophila*, all compromise organismal viability in response to DNA damaging ICL or DSB agents (Houghtaling *et al.* 2003; Marek and Bale 2006; Lee *et al.* 2007, 2010; Vinciguerra *et al.* 2010; Bretscher and Fox 2016; Ramanagoudr-Bhojappa *et al.* 2018; Yang *et al.* 2019; Germoglio *et al.* 2020; Clay *et al.* 2021). The conservation of critical FA proteins across various species emphasizes the importance of the pathway in maintaining genomic stability. This conservation necessitates further development of accessible genetic models to shed light on FA pathway mechanisms (Rodríguez and D’Andrea 2017; Niraj *et al.* 2019; Clay and Fox 2021).

Here, we employ several novel and recently developed *Drosophila* tools to study conserved roles of *fancd2.* We expand on our recent characterization of the first ever monoubiquitination-deficient *fancd2* animal model in fly development. Further, we characterize a line with 2 point mutations in *Drosophila fancd2*, one of which occurs at a highly conserved site including in humans, which we show also compromises Fancd2 DSB response function in flies. This conserved site is linked to human breast cancer metastasis and recurrence (Yates *et al.* 2017). We also introduce a monoubiquitination-deficient GFP-tagged *fancd2* transgene and show that monoubiquitination of Fancd2 is critical for its ability to form foci in response to DSBs in vivo, and to recruit the DNA repair factor Mre11. Together, these findings establish new tools for FA research and further highlight the utility of *Drosophila* to reveal in vivo-relevant roles of FA protein function.

## Materials and methods

### *Drosophila* culturing

All flies were raised at 25°C on standard media unless noted otherwise (Archon Scientific, Durham, NC, USA). For I-CreI induction, animals were heat shocked at 37°C for 90 min at the second larval instar stage. For allele frequency animal viability experiments, a TM3 balancer chromosome was used to create heterozygous animals. The following stocks were obtained from the Bloomington *Drosophila* Stock Center: *w^1118^* (#3605, used as the heterozygous control chromosome source in allele frequency experiments), *Df(3R)BSC43* (#7413), *nos-Cas9* (#54591), *nos-PhiC31*; *attP40* (#25709), *hs-I-CreI* (#6936). *Ubi-mre11* was obtained from Dr Anne Royou (Landmann *et al.* 2020). This study describes the generation of the following stocks.

### CRISPR-Cas9 editing of *Drosophila fancd2*

CRISPR-Cas9 editing was performed by Genetivision (genetivision.com). Guide RNAs, cloned into the pCDF3 expression vector (Addgene), were injected into *nos-Cas9* embryos, along with HDR templates. Guide RNA sequences were as follows.

Nested guide RNAs for first HDR event:

*fancd2* gRNA1F: GTCGTGGGGCGATGATTTGTCAC

*fancd2* gRNA1R: AAACGTGACAAATCATCGCCCCA

and

*fancd2* gRNA2F: GTCGTACGCACCGAGGCATCAAT

*fancd2* gRNA2R: AAACATTGATGCCTCGGTGCGTA

Nested guide RNAs for second HDR event:

*fancd2* gRNA3F: GTCGCCAAATCTTCGTACAGGTG

*fancd2* gRNA3R: AAACCACCTGTACGAAGATTTGG

and

*fancd2* gRNA4F: GTCGAATCACAAGCTCTGTAAAT

*fancd2* gRNA4R: AAACATTTACAGAGCTTGTGATT

In the first editing step, the HDR template contained a GFP cassette, flanked by two 1 kb *fancd2* sequences beyond the region targeted by the gRNAs. This resulted in DSBs near the gRNA sites, along with HDR from the repair template. In the second editing step, the HDR template was a 1,039-bp region spanning the edited *fancd2* gene containing the inserted GFP cassette, plus homology arms with *fancd2* sequences, including a K595R substitution. Editing was performed using a new set of nested gRNAs, which also introduced mutations in the gRNA PAM sites to prevent further Cas9-induced DSBs following insertion of the repair template (Supplementary Fig. 1a). To confirm loss of GFP during CRISPR editing, we performed PCR for the primers indicated in Supplementary Fig. 1b using short PCR cycle extension time conditions that highly favored amplification of a 490-bp product corresponding to *fancd2* without a GFP insertion, versus a longer ∼850 bp band with the insertion. We successfully amplified this 490 bp region in all 4 GFP-negative lines and failed to amplify a product from the same region in the GFP insertion line *fancd2^513ΔC^* (Supplementary Fig. 1b). As additional controls, we used these primers to amplify the 490 bp region in *w^1118^* flies and in flies heterozygous for a *fancd2* deficiency (*Df(3R)BSC43*). As expected, we see a clear 490 bp band after PCR with *w^1118^* DNA and a fainter band in *fancd2* deficiency heterozygotes (Supplementary Fig. 1b).

### *GFP-fancd2* flies

*GFP-fancd2* and *GFP-fancd2^K595R^* were synthesized and cloned into the pBID vector (Addgene) by Twist Biosciences (twistbioscience.com). Synthesized plasmids were purified with a ZymoPURE II Plasmid Midiprep Kit (Zymo Research) and sent to Model System Injections (Durham, NC, USA) for injection into *attP40* (chromosome 2L) embryos.

### Sequence analysis

PCR primer generation and sequencing of lines from CRISPR were by Eurofins (eurofinsgenomics.com). Comparison of *melanogaster* Fancd2 protein sequence with other *Drosophila* species and humans was done using NCBI BLAST. Fancd2 sequences in the *Drosophila* Genetic Reference panel (DGRP) were downloaded from PopFly (popfly.uab.cat/) and analyzed using Geneious software (geneious.com) to view translations.

### Irradiation

Larvae were irradiated as done previously (Bretscher and Fox 2016; Clay *et al.* 2021). Briefly, X-ray irradiation was performed in second instar larvae. Animals in 60 or 100 mm petri dishes with a thin layer of standard *Drosophila* food were placed in an X-RAD 160 PXI precision X-ray Irradiator (calibrated by a Dosimetrist) at the indicated dose.

### Fixed and live imaging

*Drosophila* tissues were dissected in 1× PBS and immediately fixed in 3.7% formaldehyde + 0.3% Triton-X. Immunofluorescence (IF) staining was performed as in Sawyer *et al.* (2017). Tissue was stained with DAPI at 5 μg/ml. The gamma H2AV (gH2Av) antibody was obtained from Developmental Studies Hybridoma bank and used as done previously (Bretscher and Fox 2016; Clay *et al.* 2021). All fixed images presented in figures were acquired using Zeiss 880 Airyscan Inverted Confocal using 20×/0.80 Plan-Apochromat, NA: 0.80, air and 63×/1.4 Plan-Apochromat, NA: 1.4, oil objectives, 405 nm diode, argon/2,488, and 561 nm diode lasers were used on a Zeiss Axio Observer Z1 with Definite Focus2. The system was controlled by Zeiss Zen 2.3. Adult rectum images were acquired using Zeiss AxioImager M.2 with Apotome processing using 20× objective.

Tissues were prepared for live imaging as described in previous studies (Fox *et al.* 2010; Schoenfelder *et al.* 2014; Stormo and Fox 2016, 2019; Clay *et al.* 2021). Images were acquired on spinning disk confocal (Yokogawa CSU10 scanhead) on an Olympus IX-70 inverted microscope using a 60×/1.3 NA UPlanSApo Silicon oil, 488 and 568 nm Kr-Ar laser lines for excitation, and an Andor Ixon3 897 512 electron-multiplying charge-coupled device camera. The system was controlled by MetaMorph 7.7.

### Statistics

All statistics were computed in Prism (versions 8 and 9, GraphPad, La Jolla, CA, USA). Adult papillar cell number and micronuclei frequency for animals expressing various RNAi constructs were analyzed using an unpaired *t* test (no DNA damage vs DNA damage). Focus index and fluorescence intensity measurements were analyzed using ordinary 1-way ANOVA with multiple comparisons (data points compared to no DNA damage control). *P*-values and *N* values are indicated in figure legends. Statistical notations used in figures: NS = not significant, **P ≤ 0.01, ***P ≤ 0.001.

## Results

### Generation of multiple CRISPR-Cas9 *Drosophila fancd2* alleles

Despite a high percentage of genome coverage by targeted gene disruption projects (Bellen *et al.* 2011; Li-Kroeger *et al.* 2018), there are currently zero-mutant alleles of *Drosophila fancd2* in public stock centers. Available *fancd2* genetic tools only allow whole gene knockdown/knockout by RNAi or CRISPR/Cas9. We previously used inducible RNAi to identify a role for *Drosophila* Fancd2 in response to DSBs that persist into mitosis. We reported this finding in *Drosophila* rectal papillar cells (Fox *et al.* 2010; Schoenfelder *et al.* 2014; Bretscher and Fox 2016; Stormo and Fox 2016, 2019; Cohen *et al.* 2020; Peterson *et al.* 2020), which lack apoptotic and cell cycle arrest responses to DNA damage and therefore accumulate mitotic DSBs (Bretscher and Fox 2016). To expand on these findings and examine the role of Fancd2 monoubiquitination directly, we then used CRIPSR-mediated homology-directed repair (HDR) to generate a point mutation in the endogenous *fancd2* locus at the monoubiquitinated lysine (Marek and Bale 2006) (K595, changed to R) that is conserved from human to *Drosophila* (Clay *et al.* 2021).

**Fig. 1.**
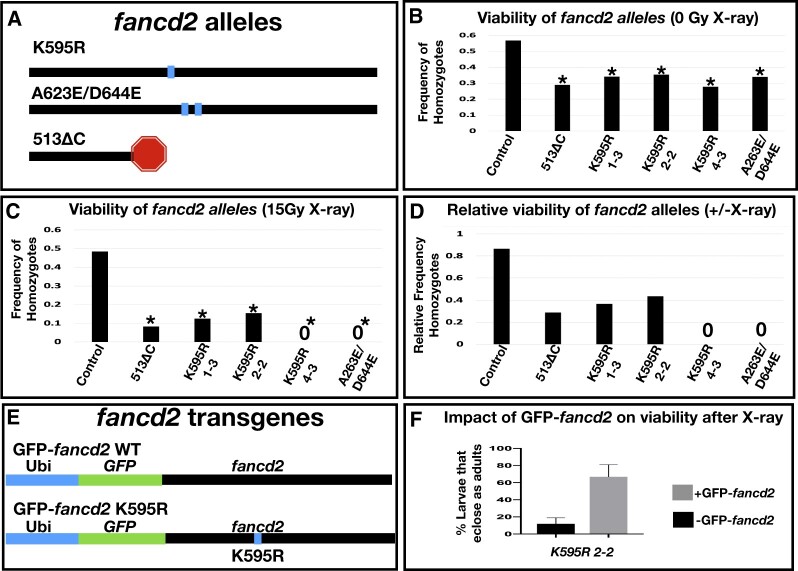
Fancd2 genetic reagents and their impact on viability with and without X-irradiation. a) Diagram of the protein level changes on *fancd2* for each indicated mutant. The horizontal line represents the *fancd2* protein. Box = point mutant, stop = truncated protein. b) The frequency of eclosed homozygotes of the indicated genotypes, from crosses with heterozygous animals. From multiple independent replicates, a total of at least 60 animals were examined per genotype. Control genotype was derived from a cross between the strain *w^1118^* and *Ly/TM3*, and for the experiment TM3 (non-Ly) animals were crossed to each other. * = significantly different from control, *P* < 0.01, Chi-squared test. c) The frequency of eclosed homozygotes of the indicated genotypes, as for panel (b), from crosses heterozygous animals where F1 progeny were irradiated (15 Gy X-ray). From multiple independent replicates, a total of at least 100 animals were irradiated per genotype. Combined number of surviving heterozygotes in each condition from all replicates (from left to right on graph): 68, 33, 7, 11, 8, 38. A “0” indicates no homozygotes survived to adulthood in any replicate. * = significantly different from control, *P* < 0.01, Chi-squared test. d) The relative frequency of homozygous animal survival for each indicated genotype. Data for each genotype represent the ratio of the value for each genotype in panel (c) divided by the same value in panel (b). e) Schematics of *GFP-fancd2* transgenic constructs with WT monoubiquitination reside and the K595R point mutation. f) Quantification of the % survival of flies that eclose as adults in *fancd2^K595R^* mutants with and without *GFP-fancd2* following 20 Gy X-ray larval irradiation.

Our editing of the K595 residue, which involved an established 2-step editing strategy (Gratz *et al.* 2013, 2014), also led to additional new *fancd2* alleles. In the first step, 2 sets of nested CRISPR/Cas9 gRNAs were designed to delete a 490-bp *fancd2* fragment containing K595 (Supplementary Fig. 1a, *Materials and* *Methods*). The resulting repair causes a Fancd2 protein truncation at amino acid 513 of the 1,478 amino acid coding sequence, caused by GFP insertion (Supplementary Fig. 1a). This mutant does not function as a reporter of Fancd2 localization as the GFP tag is not in frame and is translated separately. Hereafter, we refer to this C-terminal deletion line as *fancd2^513ΔC^* (Fig. 1a).

In the second step, a similar HDR strategy was used to replace the GFP insertion and restore the full *fancd2* gene, but now with a K595R substitution (Materials and methods). Initial confirmation of editing was confirmed by loss of GFP expression following the second editing step. We assessed the ability of the editing strategy to generate the *fancd2^K595R^* mutation using PCR and sequencing (Supplementary Fig. 1b, *Materials and* *Methods*). Four independent lines (designated #1-3, #2-2, #4-3, and #5-2) showed the expected GFP loss after expression of the second set of gRNAs (Supplementary Fig. 1b). We then sequenced the recovered PCR product from each of the 4 independent GFP lines. Three out of 4 lines contain the K595R mutation (lines #1-3, #2-2, and #4-3, Fig. 1, c and d). The #5-2 line did not contain a K595R mutation but instead contains 2 substitutions not present in the parental line: A623E and D644E (Fig. 1a and Supplementary Fig. 1c).

We were surprised to find the unintended A623E and D644E mutations from our HDR CRISPR strategy. One possibility was that this line arose from a recombination event with a homologous chromosome during editing. If so, the A623E/D644E substitutions might be present in other *Drosophila* lines. To search for support of this idea, we examined *fancd2* sequences from wild isolates in the DGRP (Huang *et al.* 2014) and from sequenced *Drosophila* species. Indeed, both A623E and D644E are present in numerous DGRP lines, indicating these are common variants in *fancd2* (Supplementary Fig. 2). In fact, E623 is more common in the DGRP than A623. Beyond *melanogaster*, an A at the position matching *melanogaster* 623 is not found in any other sequenced *Drosophila* species, whereas E is highly present. Neither an A nor an E residue matches at this portion of the protein in humans (Supplementary Fig. 2). We next examined the occurrence of D/E at Fancd2 position 644. D is much more common than E in the DGRP and is by far the predominant amino acid at this matching position in *Drosophila* species (Supplementary Fig. 2). Interestingly, D644 is conserved in humans (D604), and D to E substitution at this site is linked to breast cancer recurrence (see Discussion). As we show in the next section, *fancd2^A623E/D644E^* leads to several phenotypes also seen in *fancd2 RNAi*, *fancd2^K595R^*, and *fancd2^513ΔC^* mutants. In conclusion, through a 2-step CRISPR/Cas9 HDR strategy, we successfully generated 2 novel molecular lesions in the *Drosophila fancd2* gene: *fancd2^K595R^*, and *fancd2^513ΔC^*, and also identified *fancd2^A623E/D644E^*, a third mutant line.

### Impact of *Drosophila fancd2* mutants on viability with and without X-irradiation

Having generated the first whole animal mutant alleles of *Drosophila fancd2*, we next assessed their impact on whole animal and tissue-specific phenotypes. We first examined the viability of each *fancd2* allele by generating heterozygotes over a third chromosome balancer (*fancd2* is on the right arm of chromosome 3). From a population of heterozygous parents, we scored the relative frequency of heterozygous and homozygous F1 progeny relative to a control line heterozygous for the same balancer as the *fancd2* lines (*Materials and Methods*). All *fancd2* heterozygous lines produced viable homozygote offspring but had an under-representation of homozygous animals that eclosed compared to controls (Fig. 1b). Further, all lines retain the balancer chromosome over time. This suggests that *fancd2^K595R^*, *fancd2^A623E/D644E^*, and *fancd2^513ΔC^* are semi-lethal alleles of *fancd2.* We then assessed the ability of each allele to be maintained as a homozygous mutant stock. Except for *fancd2^K595R^*, line #4-3, homozygous animals can produce a stable fly stock. This suggests that *fancd2^K595R^* line #4-3 may have a second site mutation that impacts fertility, as other *fancd2^K595R^* lines are homozygous fertile. Taken together, these results show that, in the absence of DNA damage, all of our identified *fancd2* alleles are semi-lethal and generate viable offspring.

Given previous work in connecting *fancd2* to DSB responses, including our own work on persistent mitotic DSBs in *Drosophila* papillar cells (Bretscher and Fox 2016; Clay *et al.* 2021), we characterized the new *fancd2-*mutant alleles under conditions promoting DSBs. We induced DSBs with X-ray ionizing radiation (IR). As done previously, we irradiated at the second larval instar stage. We repeated our assay of scoring F1 progeny eclosing from a population of heterozygous parents for each allele, only this time the F1 larvae were exposed to 15 Gy IR. Irradiation had no obvious impact on frequency of homozygous control animals. In contrast, radiation substantially reduced the frequency of surviving *fancd2* homozygous progeny for all alleles (Fig. 1, c and d). This indicates that the conserved monoubiquitination residue of Fancd2, the alanine at position 623 and/or the conserved glutamic acid at position 644, and the sequences deleted in *fancd2^513ΔC^* are required for Fancd2 to properly respond to X-ray DNA damage.

To assess the specificity of these *fancd2* allele phenotypes and also study Fancd2 localization, we next generated a full-length, GFP-tagged *fancd2* rescue transgene. We cloned full-length *fancd2* as well as *fancd2* encoding a K595R mutation into a vector containing a *ubiquitin* promoter (*Materials and* *Methods*). We generated an in-frame N-terminal fusion of the *fancd2* full-length coding sequence to a codon optimized eGFP sequence. We separated GFP and *fancd2* sequences with a (GSSS)_4_ linker at the N-terminus (Fig. 1e). The vector we used also contains attB sites, allowing for phiC31-mediated integration into the genome. We integrated this construct at *attP40* on chromosome 2. Hereafter, animals with this construct are referred to as *GFP*-*fancd2* or *GFP-fancd2^K595R^.*

We examined the ability of *GFP-fancd2* to rescue the viability of *fancd2* mutants following DSBs. In this assay, we compared survival of homozygous *fancd2^K595R^* animals (line 2-2) irradiated as second instar larvae, +/− the *GFP-fancd2* transgene. The percentage of *fancd2^K595R^* homozygous larvae that survive to adulthood after 20 Gy X-ray IR increases ∼6-fold in animals also expressing *ubi-GFP-fancd2* (Fig. 1f), indicating that *GFP-fancd2* can potently rescue *fancd2^K595R^* animal survival after X-ray IR.

### Impact of *Drosophila fancd2* mutants on papillar cell viability after targeted DSB induction

*Drosophila* papillar cells lack apoptotic and cell cycle arrest responses to DSBs and enter mitosis with DSBs. However, Fancd2 and the alternative end joining regulator Pol Theta cooperate to segregate broken chromosomes during papillar mitosis, which prevents detrimental micronuclei and cell loss (Bretscher and Fox 2016; Clay *et al.* 2021). A heat-shock-inducible I-CreI endonuclease transgene (which cuts in the ribosomal DNA) amplifies the naturally occurring DSBs in papillar cells, and we have used this approach in the past to challenge papillar cells with DSBs. Using this approach, we previously showed that *fancd2 RNAi* or homozygous *fancd2^K595R^* line #2-2 causes a DSB-specific decrease in papillar cell survival following either I-Cre or IR induction, due to the formation of micronuclei from persistent mitotic DSBs (Bretscher and Fox 2016; Clay *et al.* 2021).

**Fig. 2.**
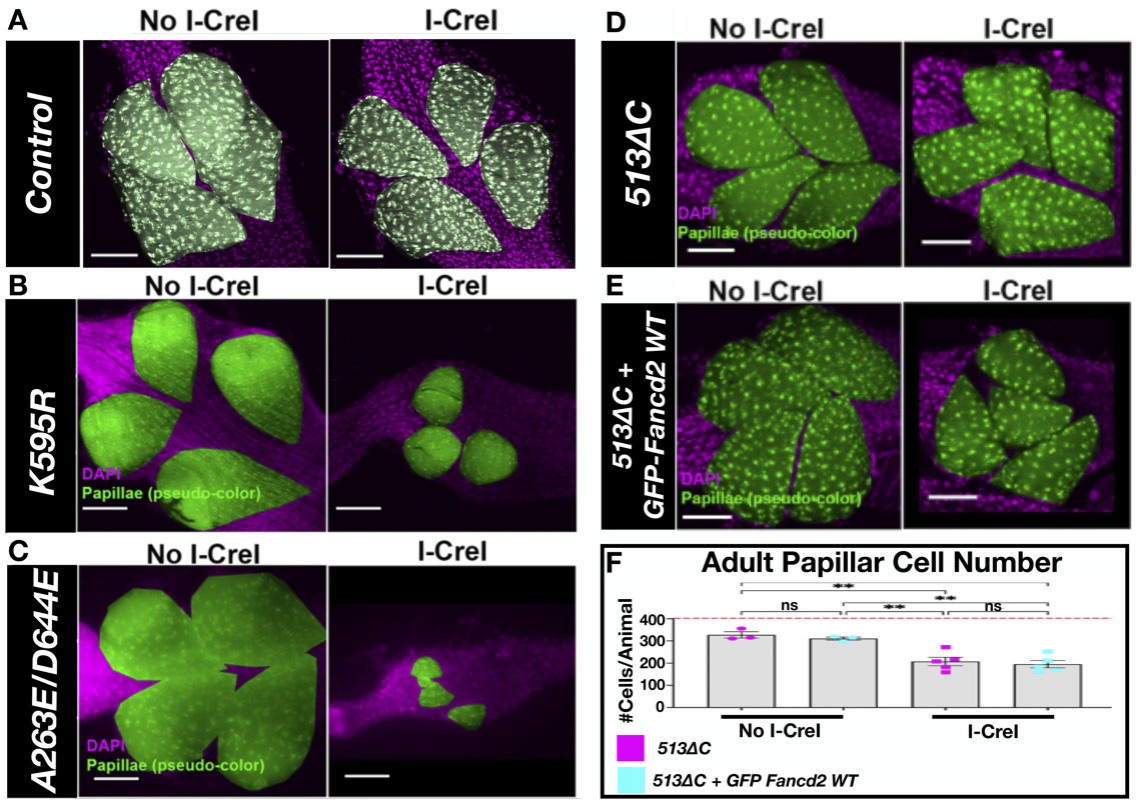
Papillar cell survival after DSB induction in *fancd2* mutants. a) Representative image of an adult rectum of control animals +/− I-Cre. Papillar cells (pseudo-colored), green; DNA (DAPI), magenta. (DNA). Scale bars = 50 μm. b) Representative image of an adult rectum of *fancd2^K595R^* (line *#*4-3) animals +/− *hs-I-CreI*. Labeling as in (a). c) Representative image of an adult rectum of *fancd2^A623E/D644E^* (line *#5-*2) animals +/− *hs-I-CreI*. Labeling as in (a). d) Representative images of adult rectums of *fancd2* deletion animals +/− *hs-I-CreI.* Labeling as in (a). e) Representative images of adult rectums of *fancd2* deletion animals +/− *hs-I-CreI* + *GFP-fancd2*. f) Quantification of adult papillar cell number in *fancd2^513ΔC^* animals +/− *hs*-*I-CreI* with and without *GFP-fancd2*. Red dashed line = expected number of papillar cells in a WT adult. Each condition has at least 2 biological replicates. Each data point represents a single animal (*N*). Statistical test: ordinary 1-way ANOVA. See Materials and methods for statistical notations.

Similar to these results, we find that an additional *fancd2^K595R^* line #4-3, and both *fancd2^A623E/D644E^* and *fancd2^513ΔC^* impact papillar cell survival after I-Cre (Fig. 2, a vs b–d). As *fancd2^513ΔC^* removes K595, the phenotype in this mutant is consistent with the role of Fancd2 monoubiquitination in DSB responses. However, the similar DSB phenotype in *fancd2^A623E/D644E^* mutants highlights a DSB-specific role for Fancd2 beyond monoubiquitination at K595.

We then determined if *GFP-fancd2* animals rescue *fancd2^513ΔC^* papillar cell phenotypes following DSBs. Compared to *fancd2^K595R^* line #4-3 and *fancd2^A623E/D644E^*, I-Cre1 expression leads to less papillar cell loss in *fancd2^513ΔC^* animals, though there is a significant cell number decrease relative to without I-Cre1 expression (Fig. 2, d and f). We find that *GFP-fancd2*; *fancd2^513ΔC^* animals still exhibit a significant decrease in adult papillar cell number following DSBs (Fig. 2, e and f). Thus, *GFP-fancd2* can substantially rescue *fancd2^K595R^* animal-level IR survival phenotypes, but not *fancd2^513ΔC^* papillar cell-level IR phenotypes. Together, these rescue experiments show that *GFP-fancd2* is sufficient to rescue some (but not all) *fancd2* mutant phenotypes.

### The conserved Fancd2 monoubiquitination lysine impacts the localization of both Fancd2 and Mre11

**Fig. 3.**
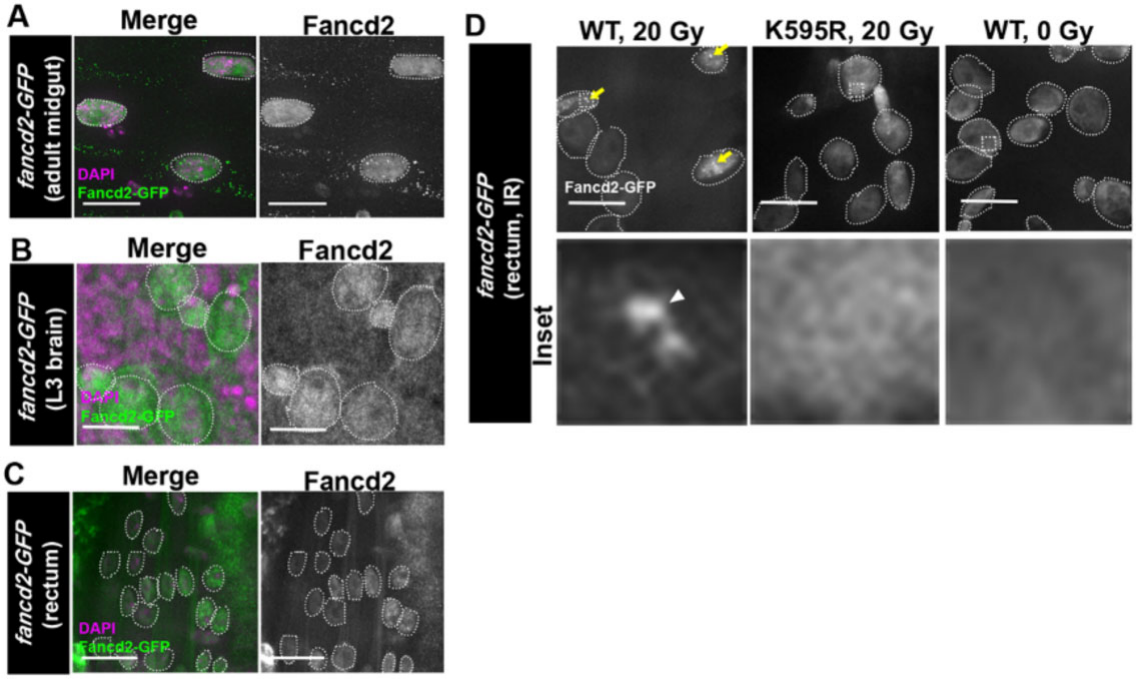
GFP-Fancd2 localization in tissues. a) Representative GFP-Fancd2 expression in the adult midgut. Enterocytes are outlined with white hatched lines. DAPI, magenta, Fancd2-GFP, green. Scale bars = 10 μm. b) Representative GFP-Fancd2 expression in the wandering third instar (L3) brain. Neuroblasts and ganglion mother cells are outlined with white hatched lines. All other labeling as in (a). c) Representative GFP-Fancd2 expression in the L3 rectum. Papillar cells are outlined with white hatched lines. All other labeling as in (a). d) Representative GFP-Fancd2 (WT and K595R) expression in the mitotic stage pupal rectum (Fox *et al.* 2010) after IR, as well as WT GFP-Fancd2 no IR control. Papillar cells are outlined with white hatched lines. GFP-Fancd2, gray. Yellow arrows = Fancd2 + foci. Hatched box = area magnified 10× in the corresponding inset below each panel. White arrowhead = micronucleus. Scale bars = 10 μm.

Given the ability of *GFP-fancd2* to rescue *fancd2^K595R^* IR-specific animal phenotypes, we examined the localization of this transgene in vivo in several cell types. In the absence of exogenous DNA damage in several tissues, GFP-Fancd2 displays pan-nuclear expression, including the adult midgut (small intestine), the developing (L3) brain, and in the developing (L3) rectum. We specifically observe an increase in GFP-Fancd2 expression in polyploid enterocytes (ECs) in the midgut (Fig. 3a). Fancd2 plays a major role in S-phase and it is possible that cells that frequently enter S-phase (endocycling cells) would have increased Fancd2 dependency (Moldovan and D’Andrea 2009; Rodríguez and D’Andrea 2017; Niraj *et al.* 2019; Peterson and Fox 2021). Additionally, neural brain progenitors also contain pan-nuclear GFP-Fancd2 WT expression (Fig. 3b). Lastly, GFP-Fancd2 displays pan-nuclear expression in papillar cells at both the larval (endocycle) and pupal (mitosis) stages (Fig. 3c, D-WT, 0 Gy).

We previously reported that GFP-Fancd2 forms foci after I-CreI expression in papillar cells (Clay *et al.* 2021). To examine whether X-ray IR causes a similar change in GFP-Fancd2 localization in this cell type, we live-imaged animals expressing GFP-Fancd2 following the second larval instar stage. As for I-CreI, IR leads to detectable Fancd2 foci (Fig. 3d—WT, 20 Gy). The similar localization change in I-CreI and IR strongly suggests that focus formation is a general property of Fancd2 localization after DSBs. To next ask if monoubiquitination of Fancd2 is required for DSB focus formation, we again subjected second instar larvae to 20 Gy X-ray IR and examined GFP-Fancd2^K595R^ localization. GFP-Fancd2^K595R^ localizes in a pan-nuclear pattern in papillar cells, regardless of irradiation (Fig. 3d—K595R, 20 Gy). This result extends our previous findings on Fancd2 localization in papillar cells with DSBs, highlighting the importance of Fancd2 a conserved monoubiquitination residue in localization to DSB foci.

**Fig. 4.**
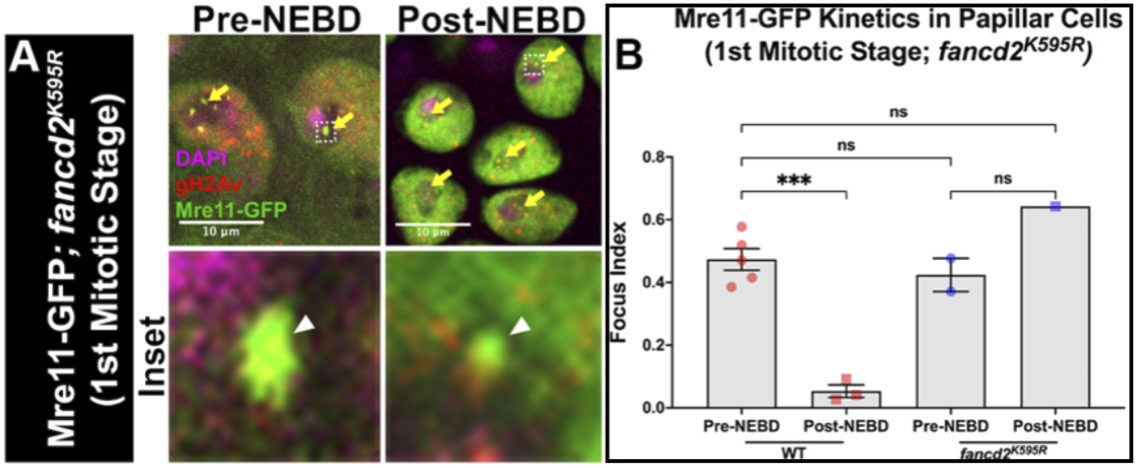
Fancd2 Lysine 595 is required for Mre11 foci removal during mitosis. a) Mre11 and gH2Av recruitment to *fancd2^K595R^* papillar cells +/− *hs*-*I-CreI*. DNA (DAPI), magenta; Mre11, green; gH2Av, red. Yellow arrows = Mre11 + gH2Av + foci. Hatched box = area magnified 10× in the corresponding inset below each panel. White arrowhead = micronucleus. Hatched outline = nuclei. Scale bars = 10 μm. NEBD = Nuclear Envelope Break Down. b) Quantification of Mre11+ gH2Av+ foci recruitment in *fancd2^K595R^* papillar cells +*hs*-*I-CreI* pre- and post- the first pupal mitotic division of the pupal stage (first mitotic stage). Focus index= average number of foci/cell in papillar cells of a given animal. Each data point represents a single animal (*N*). Statistical test: Ordinary 1-way ANOVA. See Materials and methods for statistical notations.

To understand the consequences of mis-localized, ubiquitination-deficient Fancd2, we closely examined how loss of Fancd2 monoubiquitination impacts DNA repair signaling. In our recent study, we revealed that a failure to remove the repair marker Rpa3 from DSBs underlies the DSB phenotype of *fancd2^K595R^* mutants. Further, we showed that Mre11 recruits Rpa3 to papillar DSBs. An outstanding question was whether this failure to remove Rpa3 might coincide with failure to recruit Mre11. This was an open question, given that Mre11 has distinct kinetics on papillar DSBs relative to Rpa3 (Clay *et al.* 2021), and so it remained possible that Fancd2 monoubiquitination specifically acts to remove Rpa3 and not Mre11. To test whether Fancd2 monoubiquitination is required for removal of Mre11 from papillar cell DSBs, we generated flies containing both the *fancd2^K595R^* mutation and a *ubi-mre11-GFP* transgene. Using heat-inducible I-CreI to induce DSBs during the second larval instar stage, we observe that Mre11 foci persist in papillar cells following the first mitosis (Fig. 4, a and b). This contrasts with what we observe in WT animals, in which Mre11 foci are cleared prior to Nuclear Envelope Break Down (NEBD, Fig. 4b). Taken together with our previous work, we conclude that the conserved monoubiquitination residue in Fancd2 is required for both RPA3 and Mre11 foci removal following DSBs. These data provide additional evidence of DSB-specific responses by monoubiquitination of *Drosophila* Fancd2, which involve regulation of Mre11 localization.

## Discussion

Study of the evolutionarily conserved features of FA proteins such as Fancd2 can reveal critical roles of this protein on subcellular localization, responses to various forms of DNA damage, and organismal health/disease. Here, we characterize 5 *Drosophila* Fancd2 reagents: (1) a CRISPR mutant lacking the conserved monoubiquitination residue of Fancd2 (*fancd2*^K595R^), (2) a line containing 2 amino acid substitutions that are present in natural *Drosophila* populations, one of which is at another conserved residue (*fancd2^A623E/^*^D644E^), (3) a CRISPR deletion allele resulting in a premature *fancd2* truncation, (4) a transgenic animal ubiquitously expressing a GFP-tagged WT *fancd2* rescue transgene, and (5) a transgenic animal ubiquitously expressing a GFP-tagged *fancd2* with the K595R point mutation. As we show here, these reagents are useful new tools in understanding the role of conserved Fancd2 residues in regulating genome stability.

### Roles for conserved Fancd2 point mutations in vivo

Many human FA patient mutations are point mutations (Bagby 2003), which are associated with a variety of disease phenotypes. For example, FANCD2 mutations correlate with an increased risk of head and neck squamous cell carcinoma (Chandrasekharappa *et al.* 2017) and esophageal cancer (Akbari et al. 2011). The CRISPR mutants we describe here are (to our knowledge) the first in vivo edits of conserved residues of the endogenous Fancd2 gene in any organism. We serendipitously discovered a line (*fancd2^A623E/^*^D644E^) with 2 amino acid substitutions that are common in flies. Our discovery of these substitutions highlight the possibility of repair with the homologous chromosome during CRISPR HDR as an alternative to the intended editing outcome. We note that homologous recombination occurs frequently in *Drosophila* (Rong *et al.* 2002; Brunner *et al.* 2019; Fernandez *et al.* 2019) and can even involve regions of balancer chromosomes (Miller *et al.* 2016). As we show here, like *fancd2^K595R^* mutants, *fancd2^A623E/^*^D644E^ mutants show a loss in papillar cell number following DSBs. Interestingly, in humans, *fancd2^D604E^* was found in a large-scale study of mutations linked to breast cancer metastasis and relapse (Yates *et al.* 2017). It is clear that this conserved residue is critical to Fancd2 function, and we speculate that the D644E is the substitution underlying the phenotypes we report here for the *fancd2^A623E/^*^D644E^ line.

Instead of point mutations, previous studies of Fancd2 in systems such as mice, zebrafish, *C. elegans*, and *Drosophila* have focused on complete gene knockout or knockdown. In mice, molecular null Fancd2 heterozygous animals yield homozygotes at reduced Mendelian frequencies in specific genetic backgrounds (Houghtaling *et al.* 2003; Parmar *et al.* 2010; Yang *et al.* 2019), and a *C. elegans* deletion allele has egg-laying defects (Lee *et al.* 2007, 2010). Our findings here with reduced Mendelian frequencies of homozygotes in *Drosophila* for our 2 CRISPR point mutant alleles in conserved residues, as well as the deletion allele, mirror those previous findings with whole gene knockouts*.* Upon DNA damage, Fancd2 molecular nulls in mice, zebrafish, and *C. elegans* are more sensitive to a variety of DNA damaging insults, including to radiation (Houghtaling *et al.* 2003; Lee *et al.* 2007, 2010; Ramanagoudr-Bhojappa *et al.* 2018; Yang *et al.* 2019). Our findings here suggest the conserved residues mutated in our *Drosophila* lines likely underlie these phenotypes seen with whole gene knockouts/knockdowns. As highlighted by the fact that the *Ubi-GFP-fancd2* transgene substantially rescues *fancd2*^K595R^ animal survival, but not papillar cell survival of *fancd2* deletion animals, there could likely be phenotypic differences between monoubiquitination-deficient and truncation alleles as well. Alternatively, or additionally, the difference in ability to rescue different *fancd2* phenotypes could suggest our transgene lacks endogenous sequence elements (e.g. promoter, UTRs) that are required for full rescue of all *fancd2* phenotypes.

In the future, our newly developed mutants can be further studied to identify additional phenotypes linked to conserved residues. For example, the viability and fertility of *fancd2^K595R^* mutants can facilitate future epistasis tests with other *Drosophila* repair mutants, to determine which of the major DNA repair pathways *fancd2* may regulate in a given context. We recently used *fancd2^K595R^* mutants to demonstrate that *fancd2* functions with the alternative end joining (Alt-EJ) regulator DNA Polymerase Theta in papillar cells with persistent mitotic DSBs (Clay *et al.* 2021). However, in other contexts, *fancd2* may act with regulators of other repair processes, namely homologous recombination or canonical nonhomologous end joining. Additionally, our mutants can be used to evaluate the role of conserved Fancd2 residues in processes such as recombination, replication, and repair of lesions such as ICLs. Of note, *Fancd2* null mutant mice exhibit a variety of phenotypes, including reduced body size, some degree of abnormal germ cells, eye abnormalities an increased incidence of tumors such as adenomas, and anemia, mirroring the anemia found in human FA patients (Houghtaling *et al.* 2003; Parmar *et al.* 2010; Yang *et al.* 2019). Closer examination of the new *Drosophila fancd2* mutants described here in the near future can assess more closely the contribution of, e.g. monoubiquitination, to any similar phenotypes in flies.

In addition to organismal phenotypes, the new alleles described here can be used to study Fancd2 cellular function. As we show here, the *fancd2*^K595R^ mutation results in persistent Mre11 foci during mitosis following DSBs. While K595 (human K561) is strongly linked to monoubiquitination, little is known in any model system about the region containing A623 and D644 (human D604). Our serendipitous discovery of this mutant highlights the importance of genetic reagents that are not whole gene knockouts or knockdowns in revealing mechanisms of protein regulation.

### In vivo roles of the conserved Fancd2 monoubiquitination residue in DSB repair protein localization

Low levels of Fancd2 ubiquitination can be a diagnostic indicator of FA (Pilonetto *et al.* 2009; Liu *et al.* 2013) and correlate with shorter time of disease-free survival in the context of sporadic breast cancer (Feng and Jin 2019). Monoubiquitination of vertebrate Fancd2 is sufficient for targeting to chromatin in response to DNA damaging agents such as mitomycin C (Matsushita *et al.* 2005). Here, we show this control of Fancd2 localization by a conserved monoubiquitination site occurs in vivo in *Drosophila* papillar cells in response to X-rays and I-CreI expression. We find that GFP-Fancd2, but not GFP-Fancd2^K595R^, displays pan-nuclear expression in cycling cells (mitotic and endocycling) in both developing and adult *Drosophila* tissues. Using these localization tools, future study could use live imaging to examine Fancd2-GFP kinetics, and how recruitment to Fancd2 in real time occurs relative to other factors impacted by Fancd2 loss. For example, as we show here, Mre11 is disrupted in *fancd2^K595R^* mutants.

In summary, this study highlights the power of in vivo genetics and protein localization studies to reveal conserved regulation of critical Fancd2 residues. As we suggest here, future studies in all model systems can benefit from targeted CRISPR approaches and localization studies with tagged rescuing transgenes.

## Data availability

The data underlying this article are available in the article and in its online supplementary material.

Supplemental material is available at *G3* online.

## Supplementary Material

jkac129_Supplementary_Data
