# Supplementary material for: Conserved function of Drosophila Fancd2 monoubiquitination in response to double-strand DNA breaks
Source: G3 (Bethesda). 2022 May 20;12(8):jkac129. doi: 10.1093/g3journal/jkac129 (PMC9339327; doi:10.1093/g3journal/jkac129)
Supplement: jkac129_Supplementary_Data [file jkac129_supplementary_data.zip › Suppl/Supplemental_Material_Legends_G3-2022-403474.docx]

**Supplemental Material**

**Figure S1: CRISPR-Cas9 generation of *Drosophila fancd2* alleles. A)** Two-step CRISPR homology-directed repair scheme used to generate *fancd2^K595R^* animals. The black horizontal line represents the *fancd2* gene. Location of an inserted GFP sequence, gRNA sites, introduced point mutation, and primers used for PCR are indicated. **B)** Gel electrophoresis of 490 bp region of *fancd2* amplified from primers generated indicated in panel **A**. From left to right: Ladder, line #1-3, line #5-2, line #4-3, line #2-2, *w^1118^*, *fancd2* deletion, *fancd2* deficiency/+, Ladder. The 500 bp gel location is labeled in red. **C**) Sequence alignment from NCBI BLAST for *fancd2^K595R^* (#1-3) and *fancd2^A623E/D644E^* (#5-2) lines.

**Figure S2. Frequency of amino acid substitutions in the DGRP, other *Drosophila* species, and humans at positions corresponding to *Drosophila melanogaster* Fancd2 623 and 644.** The left column lists the species examined. REF= *melanogaster* reference genome. DGRP= *melanogaster* DGRP genomes. The right two columns list the amino acid that aligns with positions 623 and 644 by NCBI Blast with *melanogaster* reference Fancd2 protein sequence. Red boxes highlight less common amino acids at each position. Black boxes highlight instances where the *melanogaster* amino acid has no match by BLAST. The percentages in parentheses indicate the frequency of occurrence of the predominant amino acid at this position within the DGRP collection.
