# Supplementary material for: Conserved function of Drosophila Fancd2 monoubiquitination in response to double-strand DNA breaks
Source: G3 (Bethesda). 2022 May 20;12(8):jkac129. doi: 10.1093/g3journal/jkac129 (PMC9339327; doi:10.1093/g3journal/jkac129)
Supplement: jkac129_Supplementary_Data [file jkac129_supplementary_data.zip › Suppl/Figure_S2_G3-2022-403474.pdf]

## Figure S2

| Species | 623 | 644 |
|---|---|---|
| *D. melanogaster REF* | A | D |
| *D. melanogaster DGRP* | E (55%) | D (72%) |
| *D. mauritiana* | E | D |
| *D. simulans* | E | D |
| *D. yakuba* | E | D |
| *D. santomea* | E | D |
| *D. erecta* | E | D |
| *D. teissieri* | E | D |
| *D. subpulchrella* | E | D |
| *D. takahashii* | E | D |
| *D. suzukii* | E | D |
| *D. pseudotakahashii* | E | D |
| *D. ficusphila* | E | D |
| *D. setifemur* | E | D |
| *D. biarmpes* | E | D |
| *D. elegans* | E | D |
| *D. bunnanda* | Q | D |
| *D. serrata* | Q | D |
| *D. rhopaloa* | E | E |
| *D. kikkawai* | Q | D |
| *D. jambulina* | Q | D |
| *D. eugracilis* | | E |
| *D. birchii* | | D |
| *H. sapiens* | | D |
