# Supplementary figures and images for: Conserved function of Drosophila Fancd2 monoubiquitination in response to double-strand DNA breaks
Source: G3 (Bethesda). 2022 May 20;12(8):jkac129. doi: 10.1093/g3journal/jkac129 (PMC9339327; doi:10.1093/g3journal/jkac129)

Figure S1

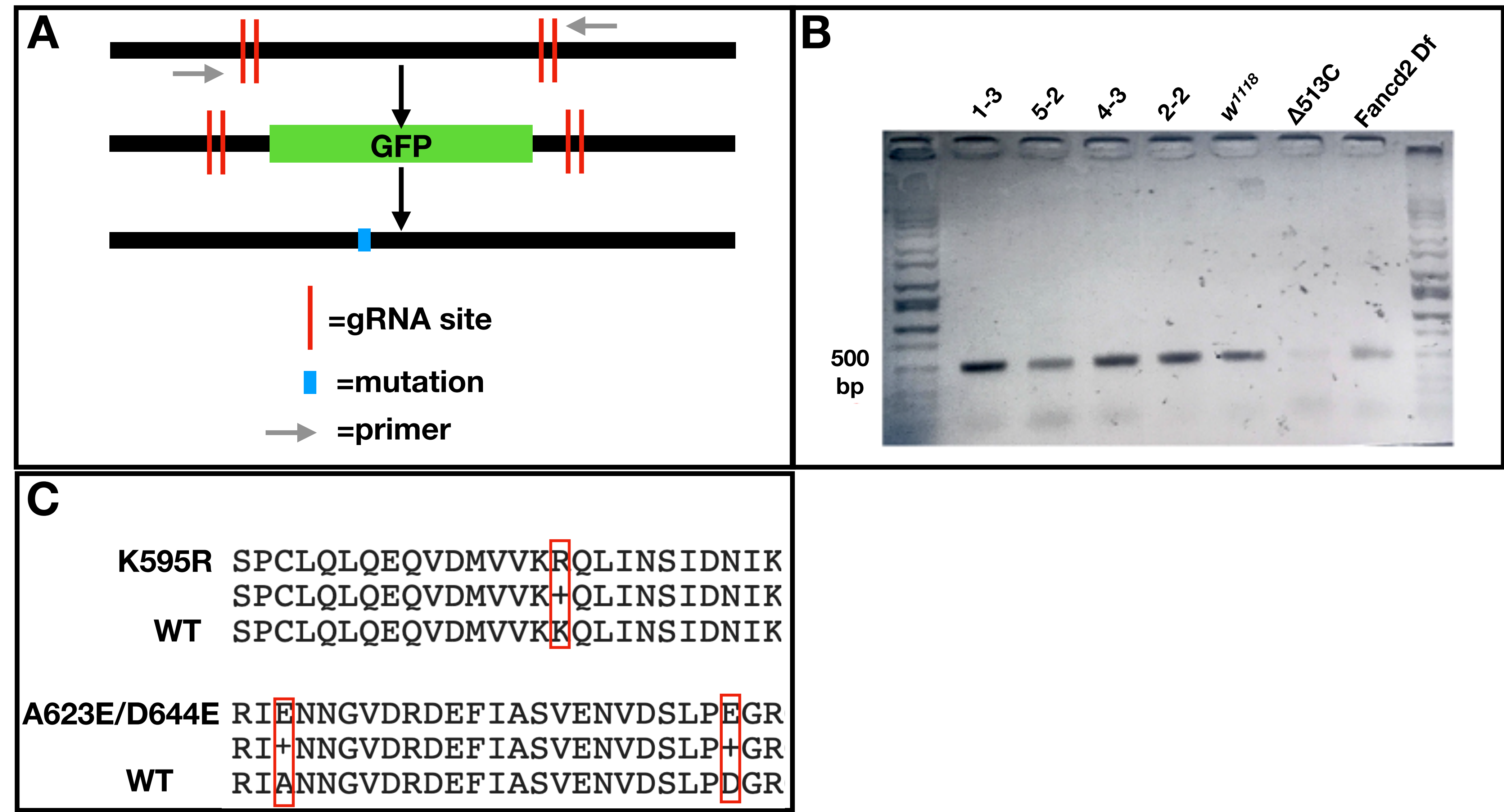

Supplement: jkac129_Supplementary_Data [file jkac129_supplementary_data.zip › Suppl/Figure_S1_G3-2022-403474.pdf]
